# Operative Time, Age, and Serum Albumin Predict Surgical Morbidity After Laparoscopic Liver Surgery

**DOI:** 10.1177/1553350621991223

**Published:** 2021-02-10

**Authors:** Daniel Heise, Jan Bednarsch, Andreas Kroh, Sandra Schipper, Roman Eickhoff, Sven Lang, Ulf Neumann, Florian Ulmer

**Affiliations:** 1 Department of Surgery and Transplantation, 39058 University Hospital RWTH Aachen, Germany; 2 Department of Surgery, 199236 Maastricht University Medical Centre (MUMC), Netherlands

**Keywords:** laparoscopic surgery, liver resection, morbidity, complications, postoperative outcome

## Abstract

*Background*. Laparoscopic liver resection (LLR) has emerged as a considerable alternative to conventional liver surgery. However, the increasing complexity of liver resection raises the incidence of postoperative complications. The aim of this study was to identify risk factors for postoperative morbidity in a monocentric cohort of patients undergoing LLR. *Methods*. All consecutive patients who underwent LLR between 2015 and 2019 at our institution were analyzed for associations between complications with demographics and clinical and operative characteristics by multivariable logistic regression analyses. *Results*. Our cohort comprised 156 patients who underwent LLR with a mean age of 60.0 ± 14.4 years. General complications and major perioperative morbidity were observed in 19.9% and 9.6% of the patients, respectively. Multivariable analysis identified age>65 years (HR = 2.56; *P* = .028) and operation time>180 minutes (HR = 4.44; *P* = .001) as significant predictors of general complications (Clavien ≥1), while albumin<4.3 g/dl (HR = 3.66; *P* = .033) and also operative time (HR = 23.72; *P* = .003) were identified as predictors of major postoperative morbidity (Clavien ≥3). *Conclusion*. Surgical morbidity is based on patient- (age and preoperative albumin) and procedure-related (operative time) characteristics. Careful patient selection is key to improve postoperative outcomes after LLR.

## Introduction

Various studies have identified laparoscopy as the standard technique for the treatment of several diseases in different surgical fields.^1,2^ A decrease in postoperative pain, a decline in hospital stay, and reduced morbidity are the major drivers of the progress of laparoscopy.^3,4^ Initially, laparoscopic approaches were not considered for liver surgery because evidence concerning technical feasibility and safety was lacking. However, further development of laparoscopic liver resection (LLR) resulted in the international consensus of 2008 that LLR is eventually recognized as a safe procedure with reasonable morbidity and mortality for minor and major liver resections when performed by hepatobiliary surgeons with extensive laparoscopic experience in specialized units.^5^ Subsequently, the indications for LLR have grown significantly in recent years. Improvements in laparoscopic tools and surgical abilities have enabled surgeons to perform more complex procedures.^6,7^ Nevertheless, the increasing complexity and extent of liver resection raises the incidence and severity of postoperative complications. Morbidity after liver resection does not only carry a significant clinical burden for the patient but it also pushes healthcare-related costs.^8^ Therefore, identifying modifiable perioperative risk factors for patients undergoing LLR is of upmost interest and might further improve the postoperative results of LLR. The aim of this study was to analyze postoperative outcome in a large monocentric cohort of patients undergoing LLR. Secondary objectives were to assess risk factors for surgical morbidity and major complications.

## Patients and Methods

This is a single-center retrospective analysis of complications after LLR resection in a consecutive cohort of patients. Institutional review board approval was obtained before analysis of the data (application no.: EK 423/19). We evaluated data of 156 patients who underwent LLR between January 2015 and August 2019 at the Department of Surgery and Transplantation of the RWTH Aachen University Hospital. Clinical data were collected prospectively in an institutional database. The indication for surgery in case of malign diseases was approved by a multidisciplinary tumor board including surgeons, hepatologists, oncologists, and radiologists. Resection extent was defined according to segmental anatomic description by Couinaud, and types of hepatectomy were classified according to Brisbane 2000 terminology.^9^ Resection of more than 3 liver segments is categorized as a major liver resection.

### Staging and Surgical Technique

All patients referred to our institution for surgical treatment were subjected to a detailed clinical workup. This included the availability of at least 1 appropriate cross-sectional imaging (gadolinium-based magnetic resonance imaging (MRI); contrast material-enhanced computed tomography (cmCT)) to determine the number, size, and location of the lesion and the presence of distant metastases, when necessary. Surgical resection was carried out in accordance with common clinical standards. Laparoscopic approach as well as the number and size of trocars were selected depending on pathologic entity, size, and localization of hepatic lesions. All resections were performed exclusively fully laparoscopic without the use of hybrid techniques. By default, the first 12 mm trocar is placed in direction or next to the resection plane to ensure optimal triangulation after placement of 2 additional 12 mm trocars. Additionally, 12 or 5 mm trocars are inserted if needed. Resection specimens were extracted through a suprapubic Pfannenstiel incision in a plastic recovery bag or via a 12 mm trocar incision. The attending surgeon was positioned between the patient’s legs who is in a left tilted supine position (French position). The pneumoperitoneum was preserved by 12 mmHg intraperitoneal pressure. Intrahepatic lesions were routinely located by laparoscopic ultrasound. Parenchymal transection was commonly performed by Thunderbeat® (Olympus K.K., Tokyo, Japan) or Harmonic Ace® (Ethicon Inc, Somerville, New Jersey, USA). If necessary, laparoscopic ultrasonic surgical aspirator (CUSA, Integra LifeSciences, New Jersey, United States) was chosen for deeper parenchymal transection close to major vascular structures. Vascular staplers (Echelon, Ethicon, Somerville, New Jersey, United States) were used for the dissection of large vessels and bile ducts.

### Statistical Analysis

The primary outcome parameter in this study was the occurrence of major perioperative morbidity defined as complications rated Clavien-Dindo ≥ 3 according to the Clavien-Dindo scale.^10^ The secondary end point was the occurrence of general complications, which was defined as any postoperative complication (Clavien-Dindo ≥ 1).^10^ Data derived from continuous variables are presented as mean and standard deviation. Associations between perioperative variables and the primary or secondary end point were assessed by means of binary logistic regression. Variables being statistically significant in univariate analysis were transferred into a multivariable model and analyzed with multivariable binary logistic regressions. For this purpose, nominal and categorial data were recoded into scaled dummy variables. The level of significance was set to P < .05, and *P*-values are given for two-sided testing. Analyses were performed using SPSS Statistics 24 (IBM Corp, Armonk, New York, USA).

## Results

### Patient Characteristics

We analyzed 156 patients who underwent LLR at our institution between January 2015 and August 2019 with a mean age of 60.0 ± 14.4 years. 43.9% of the patients were male, and mean body mass index (BMI) was 26.1 ± 5.5 kg/m^2^. The primary diagnosis was benign in 36 (23.1%) of the patients. Among patients with malignant tumors, 56 (35.9%) had colorectal liver metastasis (CLM), 32 (20.5%) hepatocellular carcinoma (HCC), 10 (6.4%) intrahepatic cholangiocellular carcinoma (iCC), and 22 (14.1%) other liver metastasis (LM). A subset of 20 (12.8%) patients presented with liver cirrhosis. 39 (25%) patients underwent a major liver resection. The mean operative time was 190 ± 100 minutes. The range of procedures included 34 (21.8%) atypical resections, 24 (15.4%) segmentectomies, 59 (37.8%) bisegmentectomies, 4 (3.6%) left hemihepatectomies, 29 (18.6%) right hemihepatectomies, 3 (1.9%) extended left hemihepatectomies, and 3 (1.9%) extended right hemihepatectomies. Intraoperative conversion to an open procedure was necessary in 8 (5.1%) cases, most commonly due to difficult access to the particular lesion or intraoperative hemorrhage not controllable by the laparoscopic technique. 24 (15.4%) patients needed intraoperative blood transfusion. Mean intensive care stay was .71 ± .8 days. More details regarding demographics, clinical characteristics, and operative data are shown in Table 1.

**Table 1.** Clinical and Perioperative Characteristics.

Demographic (n = 156)	Mean ± SD
Gender, m/f (%)	67 (42.9)/89 (57.1)
Age (years)	60.0 ± 14.4
BMI (kg/m^2^)	26.1 ± 5.5
Diagnosis, n (%)	
CRLM	56 (35.9)
HCC	32 (20.5)
iCC	10 (6.4)
Other LM	22 (14.1)
Benign	36 (23.1)
ASA, n (%)	
I	10 (6.4)
II	73 (46.8)
III	65 (41.7)
IV	8 (5.1)
Previous abdominal surgery, n (%)	47 (30.1)

Clinical characteristic	Mean ± SD
Steatosis, n (%)	28 (17.9)
Fibrosis, n (%)	41 (26.3)
Cirrhosis, n (%)	20 (12.8)
Albumin (g/dl)	4.5 ± 2.6
GGT (U/l)	89.7 ± 114.0
Total bilirubin (mg/dl)	.4 ± .3
Platelet count (/nl)	267.2 ± 94.7
Alkaline phosphatase (U/l)	91.7 ± 47.9
INR	1.0 ± .1
Hemoglobin (g/dl)	13.1 ± 1.6

Operative data	Mean ± SD
Major resection, n (%)	39 (25.0)
Operative time (minutes)	190 ± 100
Operative procedure, n (%)	
Atypical	34 (21.8)
Segmentectomy	24 (15.4)
Bisegmentectomy	59 (37.8)
Left hepatectomy	4 (2.6)
Right hepatectomy	29 (18.6)
Extended left hepatectomy	3 (1.9)
Extended right hepatectomy	3 (1.9)
Conversion	8 (5.1)
Intraoperative blood transfusion	24 (15.4)

Postoperative data	Mean ± SD
Intensive care, days	.71 ± .8	
Postoperative complications, n (%)	
No complications	125 (80.1)
Clavien-Dindo I	8 (5.1)
Clavien-Dindo II	8 (5.1)
Clavien-Dindo IIIa	8 (5.1)
Clavien-Dindo IIIb	2 (1.3)
Clavien-Dindo IVa	2 (1.3)
Clavien-Dindo IVb	0
Clavien-Dindo V	3 (1.9)
Biliary leakage		4 (2.6)
Liver failure		3 (1.9)
Wound infection		3 (1.9)
Pneumonia		5 (3.2)

Note: Data presented as mean and standard deviation if not noted otherwise.

Abbreviations: BMI = body mass index; GGT = gamma-glutamyltransferase; INR = international normalized ratio; ASA = American Society of Anesthesiologists classification. CRLM = colorectal liver metastasis; HCC = hepatocellular carcinoma; iCC = intrahepatic cholangiocellular carcinoma; LM = liver metastasis.

### Complications

The majority of patients (125, 80.1%) underwent LLR without any complication (Table 1). No intraoperative mortality occurred. Overall morbidity, defined as the occurrence of any postoperative complication (Clavien-Dindo ≥1), was observed in 31 (19.9%) patients. Major morbidity (Clavien-Dindo ≥ 3) occurred in 15 (9.6%) of the patients. Most frequent major complications were pneumonia (n = 5), biliary leakage (n = 4), liver failure (n = 3), and deep wound infection (n = 3). A total of 3 (1.9%) patients deceased in the postoperative course. One patient died of sudden asphyxia due to postoperative aspiration after right hemihepatectomy and extensive laparoscopic adhesiolysis. Two patients with HCC and liver cirrhosis died from postoperative liver failure after development of septic pneumonia.

### Univariate and Multivariable Analysis of Postoperative Morbidity

A univariate binary logistic regression was carried out for postoperative morbidity (Clavien-Dindo ≥1) including all available pre- and intraoperative variables (Table 2). In our cohort, previous abdominal surgery (HR = 2.27; *P* = .048), major resection (HR = 3.95; *P* = .001), conversion (HR = 4.48; *P* = .042), age >65 years (HR = 2.55, *P* = .22), operation time >180 minutes (HR = 4.35; *P* = .001), and preoperative albumin <4.3 g/dl (HR = 2.24; *P* = .048) were associated with postoperative complications (Table 2). These variables were subsequently included in a multivariable binary logistic regression model which determined age >65 years (HR = 2.56; *P* = .028) and operation time >180 minutes (HR = 4.44; *P* = .001) as significant predictors of postoperative morbidity (Table 3). A similar analysis on major postoperative morbidity (Clavien-Dindo ≥ 3) was also carried out. This analysis showed a significant association of major resection (HR = 7.72; *P* < .001), conversion (HR = 6.80; *P* = .015), intraoperative blood transfusion (HR = 4.56; *P* = .009), operation time >180 minutes (HR = 23.25; *P* = .003), hemoglobin >13 g/dl (HR = .28; *P* = .035), and preoperative albumin <4.3 g/dl (HR = 3.53; *P* = .028) with major postoperative complications (Table 2). These variables were also included in the corresponding multivariable binary logistic regression model which determined preoperative albumin <4.3 g/dl (HR = 3.66; *P* = .033) and operative time >180 minutes (HR = 23.72; *P* = .003) as independent predictors of major postoperative morbidity (Table 4).

**Table 2.** Univariable Analysis of Perioperative Morbidity.

	n	Major Morbidity (Clavien-Dindo ≥ 3)			Morbidity (Clavien-Dindo ≥ 1)		
		Hazard Ratio	95% CI	*P* Value	Hazard Ratio	95% CI	*P* Value
Tumor				.998			.059
Malign	120						
Benign	36						
ASA				.114			.074
≤II	83						
>II	73						
Gender				.396			.781
Male	67						
Female	89						
Steatosis				.828			.768
Present	28						
Absent	128						
Fibrosis				.211			.197
Present	41						
Absent	115						
Cirrhosis				.104			.076
Present	20						
Absent	136						
Previous abdominal surgery				.155			**.048**
Yes	47				2.27	1.01-5.11	
No	108				1		
Resection extent				**<.001**			**.001**
Major	39	7.72	2.45-24.36		3.95	1,72-9.08	
Minor	117	1			1		
Conversion				**.015**			**.042**
Yes	8	6.80	1.45-31.98		4.48	1.05-19.05	
No	148	1			1		
Intraoperative blood transfusion				**.009**			.078
Yes	24	4.56	1.45-14.32				
No	132	1					
Intensive care				.135			.110
Yes	96						
No	60						
Age (years)				.263			**.022**
≤65	94				1		
>65	62				2.55	1.14-5.69	
BMI				.845			.496
≤25	72						
>25	84						
Operation time (minutes)				**.003**			**.001**
≤180	89	23.25	2.97-181.87		1		
>180	67				4.35	1.84-10.24	
Alkaline phosphatase (U/l)				.068			.163
≤80	78						
>80	78						
GGT (U/l)				.061			.496
≤46	79	3.13	.95				
>46	77	1					
Hemoglobin (g/dl)				**.035**			.062
≤13	72	1					
>13	84	.28	.08-.91				
Platelet count (/nl)				.580			.839
≤250	73						
>250	83						
Total bilirubin (mg/dl)				.239			.286
≤.3	53						
>.3	103						
INR				.957			.290
≤1	92						
>1	63						
Albumin (g/dl)				**.028**			**.048**
≤4.3	61	3.53	1.14-10.90		2.24	1.01-2.24	
>4.3	95	1			1		

Note: Various parameters are associated with major and general postoperative morbidity. Hazard ratios are shown for statistically significant variables.

Abbreviations: ASA = American Society of Anesthesiologists classification; BMI = body mass index; GGT = gamma-glutamyltransferase; INR = international normalized ratio.

**Table 3.** Multivariable Binary Logistic Regression of Perioperative Morbidity.

Variable	Morbidity (Clavien-Dindo ≥ 1)		
	Hazard Ratio	95% CI	*P* Value
Age (years)			**.028**
≤65	1		
>65	2.56	1.11-5.93	
Albumin (g/dl)			.064
≤4.3			
>4.3			
Previous abdominal surgery			.122
No			
Yes			
Resection extent			.152
Major			
Minor			
Conversion			.198
Yes			
No			
Operation time (minutes)			**.001**
≤180	1		
>180	4.44	1.88-10.49	

Note: All variables showing statistical significance in univariate binary logistic regression were included in a multivariable logistic regression. Hazard ratios are shown for statistically significant variables.

**Table 4.** Multivariable Binary Logistic Regression of Major Perioperative Morbidity.

Variable	Morbidity (Clavien*-*Dindo ≥ 3)		
	Hazard Ratio	95% CI	*P* Value
Albumin (g/dl)			**.033**
≤4.3	3.66	1.11-12.08	
>4.3	1		
Hemoglobin (g/dl)			.095
≤13			
>13			
Resection extent			.176
Major			
Minor			
Conversion			.136
Yes			
No			
Operation time (minutes)			**.003**
≤180	1		
>180	23.72	2.99-187.88	
Intraoperative blood transfusion			.108
Yes			
No			

Note: All variables showing statistical significance in univariate binary logistic regression were included in a multivariable logistic regression. Hazard ratios are shown for statistically significant variables.

## Discussion

LLR was first reported more than 20 years ago, when French pioneers around Cherqui et al^11^ published their first series of surgeries. Since then, a number of series have been published, mainly by early adopters from the United States, Europe, and Asia.^12-15^ Although the surgical community was initially restraint, LLR in hands of experienced surgeons is nowadays considered a safe and feasible procedure. However, the procedure has still not gained widespread acceptance in several countries, including Germany, and has so far mainly been used in a limited number of high-volume hepato-pancreato-biliary (HPB) centers. LLR remains a technical challenge, especially for major liver resections and bears a significant learning curve.^16^ The first laparoscopic major hepatectomy was published in 1998, followed by the first case series in 2004.^17,18^ Early publications displayed a high conversion rate of up to 26% in extended resections, particularly due to uncontrollable hemorrhage.^19^ As a result, a number of hybrid techniques such as laparoscopic or hand-assisted procedures are described in the literature, which were mainly used during the implementation phase.^20^ In our cohort, liver resections were performed exclusively using a fully laparoscopic technique without the use of hybrid techniques. Regarding effectiveness of LLR for malignant indications, there are no differences in disease-free or overall survival compared to open hepatectomy.^3,21-23^ Furthermore, a number of publications show advantages for laparoscopic hepatectomies regarding blood loss, duration of hospitalization, and complications.^24-26^ A review by Kasai et al^27^ showed that LLR was superior to open hepatectomy in terms of minor complications, whereas no significant difference with respect to major complications could be demonstrated. The presented data add valuable aspects to the current literature as the focus was on assessing risk factors for increased surgical morbidity and major complications.

Our results are further based on a high-risk cohort since about half our patients were classified as the American Society of Anesthesiologists (ASA) III or higher. In many studies, patients are selected and the proportion of ASA I/II is up to 80%.^28-30^ In addition to the significant multi-morbidity of our patients, 26.3% presented with liver fibrosis and 12.8% with liver cirrhosis.

Major resections of at least 4 liver segments were performed in 25% of the cases. Whenever technically feasible, a parenchyma-sparing surgery was undertaken as patients are known to be more eligible for repeated surgery and, therefore, might have a better overall prognosis than patients who underwent major hepatectomies.^31^

The vast majority of the patients (80.1%) underwent surgery without complications. Overall morbidity was observed in 19.9% of the cases and major morbidity (Clavien-Dindo ≥ 3) occurred in 9.6% which is comparable to the published literature, despite the significant proportion of multimorbid patients (ASA ≥ III) in our cohort.^32^ In the present study, 3 patients deceased in the direct postoperative course (1.9%). A comparable mortality rate has already been demonstrated in other published series.^33^ Also, the overall mortality might be explained by our relatively morbid patients. Assessment of risk factors for the occurrence of any postoperative morbidity (Clavien-Dindo ≥ 1) identified age >65 years and operation time >180 minutes as significant predictors. It is noticeable that in the univariate analysis, the resection extent (major/minor) was still significant but in contrast to the operation time >180 minutes not in the multivariate analysis. This might be explained by the utilization of complex atypical or multiple LLR in our cohort, which require prolonged operative time and are prone for postoperative complications compared to standard anatomical major resection. Identification of age >65 years as a risk factor is in line with the findings reported in a meta-analysis by Chen et al, which also shows a slightly increased rate of minor complications in elderly patients vs non-elderly, however, without being statistically significant.^34^ The advantage of the minimally invasive approach seems to reduce with higher age. Subgroup analysis within a multicenter study by Martinez-Cecilia et al comparing elderlies undergoing hepatectomy for colorectal liver metastases showed that (major) complications for patients >80 years were comparable in the open and laparoscopic group.^35^ Nomi et al showed the same for minor complications in the age-group >80 years.^36^

The occurrence of major postoperative morbidity (Clavien-Dindo ≥ 3) after LLR was predicted by preoperative albumin <4.3 g/dl (HR = 3.66; *P* = .033) and also prolonged operative time (HR = 23.72; *P* = .003). A direct relationship between operative time and risk of postoperative pulmonary and infectious complications has been shown by a multivariate analysis for open hepatic resections.^37^ Tranchart et al found an increase of postoperative complications by 60% with each additional operative hour during LLR.^38^ They concluded that operative time should always be assessed before and during LLR. However, we think it is very difficult to define an exact time cutoff where the surgeon should think about converting. An ROC analysis in the abovementioned study indicated a better balance between specificity and sensitivity of predicting a postoperative complication after approximately 200 minutes of surgery and therefore concluded that conversion should be considered if a surgeon encounters persistent difficulty to progress after 3 hours operative time. Furthermore, an even more intensive postoperative assessment with regard to complications should be carried out in patients with extended operating time.

In line with our findings, a low serum albumin level has already been identified as an independent risk factor for postoperative complications in open liver surgery, especially for postoperative bile leakage, by several studies.^39-41^ Low albumin levels are commonly found in malnutrition patients and are associated with a series of physiological derangements that may lead to postoperative complications. However, the exact pathophysiology of this relationship is not clear. A review article by Kim et al fails to show a direct cause and effect between low albumin levels per se and adverse outcomes.^42^ They conclude that interventions designed solely to correct preoperative hypoalbuminemia, in particular intravenous albumin infusion, do little to change the patient’s course of hospitalization. In our perspective, a low albumin level at admission is mainly of prognostic value for the surgeon.

Our analysis has certain limitations that need to be discussed. First, our results are based on a single-center cohort analyzed in a retrospective fashion. Thus, the obtained results represent our individual technical approach to LLR and our clinical decision-making in benign or malign liver disease. However, due to the technical varieties in the LLR technique among different centers and diverse clinical standards regarding hybrid approaches or various dissection methods, a multicenter analysis with a higher sample size would also be biased in terms of the largely different surgical techniques. For this reason, we consider a homogenous surgical approach within our cohort as a strength of the analysis.

In conclusion, LLR appears to be safe in experienced high-volume centers. Careful patient selection with respect to patients’ age and preoperative albumin is key to minimize postoperative complications and improve perioperative results.

## Appendix

### Abbreviations

ASA: American society of anesthesiologists
LLR: laparoscopic liver resection
HR: hazard ratio
MRI: gadolinium-based magnetic resonance imaging
cmCT: contrast-material enhanced computed tomography
CUSA: laparoscopic ultrasonic surgical aspirator
BMI: body-mass-index
HPB: hepato-pancreato-biliary
CRLM: colorectal liver metastasis
HCC: hepatocellular carcinoma
iCC: intrahepatic cholangiocellular carcinoma
LM: liver metastasis
GGT: gamma-glutamyl transferase
INR: international normalized ratio
